# Effect of two in-office bleaching agents-35% hydrogen peroxide and 37% carbamide peroxide-on enamel microhardness: An *in vitro* study

**DOI:** 10.6026/973206300214368

**Published:** 2025-12-15

**Authors:** Thouseef C.H, Dilip Katakam, Vijay Shekhar, Manpreet Kaur, Mohan Kumar Reddy Sirigiri, Shamurailatpam Priyadarshini, Sneha Das

**Affiliations:** 1 Department of Conservative Dentistry and Endodontics, Educare Institute of Dental Sciences, Chattiparambu, Malappuram, Kerala, India; 2 Department of Conservative Dentistry and Endodontics, ESIC Dental College and Hospital, Kalaburagi, Karnataka, India; 3 Department of Dentistry, Nalanda Medical College Hospital, Agamkuan, Patna, Bihar, India; 4 Christian Dental College and Hospital, Ludhiana, Punjab, India; 5 Department of Conservative Dentistry and Endodontics, CKS Teja Institute of Dental Sciences and Research, Tirupati, Andhra Pradesh, India; 6 Department of Conservative Dentistry and Endodontics, Dental College, RIMS, Imphal, Manipur, India; 7 Department of Public Health Dentistry, Late Shri Yashwantrao Chavan Memorial Medical and Rural Development Foundation's Dental College and Hospital, Ahmednagar, Maharashtra, India

**Keywords:** Tooth bleaching, enamel microhardness, hydrogen peroxide, carbamide peroxide, *in vitro* techniques

## Abstract

Tooth discoloration frequently drives patients to seek in-office bleaching procedures, which commonly use high-concentration peroxide agents. This *in vitro* study evaluated the effect of two chairside bleaching formulations-35% hydrogen peroxide and 37% carbamide peroxide-on enamel microhardness. Hence, thirty premolars were assigned to control, hydrogen peroxide, or carbamide peroxide groups, and microhardness was measured before and after bleaching. Both bleaching agents caused significant reductions in enamel microhardness compared with the control group, although no significant difference was observed between the two peroxide systems. These findings indicate that in-office bleaching agents can adversely affect enamel hardness, highlighting the importance of post-bleaching remineralization strategies for clinical safety.

## Background:

Tooth discoloration is a common esthetic concern leading to increased demand for bleaching procedures [1]. In-office bleaching techniques, often employing high concentrations of hydrogen peroxide (HP) or carbamide peroxide (CP), are favored for their rapid and predictable results [2]. These oxidizing agents penetrate enamel and dentin, breaking down chromogenic substances responsible for staining [3]. However, bleaching may have adverse effects on dental hard tissues. Laboratory studies indicate that high-concentration bleaching agents can cause alterations in enamel morphology, roughness, and mineral content [4]. One critical parameter reflecting these changes is enamel microhardness, which directly relates to mineral integrity and resistance to wear [5]. Previous studies have reported varying outcomes. Some have observed significant microhardness reduction, whereas others noted no clinically relevant effects, possibly due to differences in agent concentration, exposure time, and experimental design [6]. Understanding these effects is essential to balance esthetic demands with long-term tooth integrity. It was hypothesized that bleaching would reduce enamel microhardness significantly compared to control specimens [7]. Therefore, it is of interest to evaluate the effect of two commonly used in-office bleaching agents on enamel microhardness *in vitro*.

## Materials and Methods:

Sample selection:

[1] Thirty extracted human premolars, free of caries, cracks, or restorations, were collected.

[2] Teeth were cleaned, stored in distilled water at 4°C, and used within one month of extraction.

## Experimental groups:

Teeth were randomly assigned into three groups (n=10 each):

[1] Group I (Control): Stored in artificial saliva without bleaching.

[2] Group II (35% Hydrogen Peroxide): Treated with in-office bleaching gel (35% HP).

[3] Group III (37% Carbamide Peroxide): Treated with in-office bleaching gel (37% CP).

## Bleaching protocol:

[1] Gels were applied according to manufacturer's instructions.

[2] Each application lasted 15 minutes, repeated three times, simulating a standard clinical session.

[3] Post-treatment, samples were rinsed and stored in artificial saliva at 37°C.

## Microhardness testing:

[1] Buccal enamel surfaces were flattened and polished using silicon carbide papers.

[2] Baseline microhardness was measured using a Vickers microhardness tester with 100 g load for 15 seconds.

[3] Post-bleaching microhardness was measured at identical sites.

[4] Mean values were calculated for each group.

## Statistical analysis:

[1] Data were analyzed using SPSS software.

[2] Paired t-tests compared baseline and post-bleaching values within groups.

[3] One-way ANOVA with post hoc Tukey test compared changes among groups.

[4] Significance was set at p < 0.05.

## Results:

Table 1 (see PDF) demonstrates that enamel microhardness in the control group remained stable, validating the reliability of experimental conditions. Conversely, both bleaching groups exhibited significant reductions. The 35% hydrogen peroxide group recorded the highest decrease (10.4%), while the 37% carbamide peroxide group showed a slightly lower reduction (8.2%). These findings confirm that in-office bleaching agents adversely impact enamel microhardness, with hydrogen peroxide producing a marginally stronger effect compared to carbamide peroxide, although both changes were clinically significant. Table 2 (see PDF) highlights the intergroup comparisons. Both bleaching groups differed significantly from the control group, confirming the bleaching agents as the cause of enamel hardness reduction. However, the difference between hydrogen peroxide and carbamide peroxide groups was not statistically significant (p = 0.08). This indicates that although hydrogen peroxide showed numerically greater hardness reduction, the two agents produced comparable adverse effects on enamel microhardness when applied under identical in-office bleaching conditions.

## Discussion:

This study confirmed that in-office bleaching agents significantly reduce enamel microhardness, consistent with the proposed hypothesis [8]. The reduction likely results from oxidative degradation of the enamel matrix and demineralization due to the acidic pH of bleaching gels [9]. Although hydrogen peroxide caused a greater numerical reduction in hardness compared with carbamide peroxide, the difference was not statistically significant. This suggests that concentration and exposure duration are more influential than the specific bleaching agent used [10]. Both agents demonstrated comparable detrimental effects under controlled conditions [11]. The present findings align with earlier laboratory studies reporting bleaching-induced demineralization and loss of enamel integrity [12]. In contrast, clinical trials often show minimal adverse effects, as saliva, fluoride, and dietary minerals help remineralize enamel and buffer acidity [13]. These discrepancies highlight the limitations of *in vitro* models, which lack protective oral factors [14]. Future research should assess long-term outcomes, especially when bleaching is combined with remineralizing strategies such as fluoride, casein phosphopeptide-amorphous calcium phosphate, or nano-hydroxyapatite, which may mitigate hardness reduction and preserve tooth structure [15].

## Conclusion:

In-office bleaching significantly reduced enamel microhardness compared with unbleached controls. Both 35% hydrogen peroxide and 37% carbamide peroxide caused similar adverse effects, with no statistically significant difference between them. Clinicians should consider post-bleaching remineralizing measures to counteract enamel alterations and ensure long-term safety of bleaching procedures.
